# SEURAT: Visual analytics for the integrated analysis of microarray data

**DOI:** 10.1186/1755-8794-3-21

**Published:** 2010-06-03

**Authors:** Alexander Gribov, Martin Sill, Sonja Lück, Frank Rücker, Konstanze Döhner, Lars Bullinger, Axel Benner, Antony Unwin

**Affiliations:** 1 Department of Computer Oriented Statistics and Data Analysis, University of Augsburg, Universitätsstr. 14, 86159 Augsburg, Germany; 2 Division of Biostatistics, German Cancer Research Center, Im Neuenheimer Feld 280, 69120 Heidelberg, Germany; 3 Department of Internal Medicine III, University Hospital of Ulm, Albert-Einstein-Allee 23, D-89081 Ulm, Germany

## Abstract

**Background:**

In translational cancer research, gene expression data is collected together with clinical data and genomic data arising from other chip based high throughput technologies. Software tools for the joint analysis of such high dimensional data sets together with clinical data are required.

**Results:**

We have developed an open source software tool which provides interactive visualization capability for the integrated analysis of high-dimensional gene expression data together with associated clinical data, array CGH data and SNP array data. The different data types are organized by a comprehensive data manager. Interactive tools are provided for all graphics: heatmaps, dendrograms, barcharts, histograms, eventcharts and a chromosome browser, which displays genetic variations along the genome. All graphics are dynamic and fully linked so that any object selected in a graphic will be highlighted in all other graphics. For exploratory data analysis the software provides unsupervised data analytics like clustering, seriation algorithms and biclustering algorithms.

**Conclusions:**

The SEURAT software meets the growing needs of researchers to perform joint analysis of gene expression, genomical and clinical data.

## Background

The rapid development of microarray technologies in recent years has led to the possibility of acquiring a large spectrum of different molecular data types. In translational cancer research, gene expression data are usually collected together with additional clinical information and genomic data from other high throughput technologies such as microarray-based comparative genomic hybridization (array CGH) or SNP (single nucleotide polymorphism) arrays. The availability of these related, mostly high-dimensional data sets calls for software tools which can analyze them all together in an integrated fashion. Currently there is a lack of such applications that enable exploratory analysis of integrated data sets. Most visualization and clustering tools are limited in their ability to handle gene expression, genomic and clinical data together. To our knowledge only a few software tools are able to perform an integrated analysis.

The VAMP software [1] is able to visualize genomic gain and loss information together with gene expression data. The focus of VAMP is on the comparison of the genomic information between tumors and thus all data types are displayed along the physical position in the genome. It is not possible to reorder the gene expression data according to the expression patterns and clustering algorithms can only be applied to cluster different tumors. A single graphic allows the display of additional clinical data by a simple color code and this representation is limited to categorical variables. In addition the graphics are not linked, so that each graphic has to be interpreted separately.

Other tools able to visualize gene expression data together with genetic variations and other molecular data types like RNAi data and methylation data are the Integrative Genomic Viewer (IGV) [2] developed by the Broad Institute and the Integrated Genome Browser [3]. These tools organize the different data types in the form of tracks within a browser window similar to the well known UCSC Genome Browser. The different data types are displayed one below the other along the physical positions of the genome. This visualization allows the user to examine relations between different molecular data at specific known genomic locations, but it is impossible to reveal new trans-regulative relations. Furthermore, with an increasing number of subjects and molecular data types the comparison of the many tracks becomes complicated. IGV additionally offers the possibility of aligning clinical data using color codes. For continuous data and especially for time to event data like survival times such a representation is not sufficient.

Besides these open source software solutions, some proprietary software tools are able to perform an integrated analysis, e.g., the Genomic Workbench (Agilent Technologies, Santa Clara, California) or Acuity (Enterprise Microarray Informatics). However, although they can handle the different data types, visualizations are limited to stand alone graphics, not linked to other displays such as clustering results or summary statistics of clinical variables. In order to reveal new biologically meaningful relations possibly hidden inside the different data sets, we follow the philosophy of exploratory data analysis [4]. Our approach to this problem was to develop open source software capable of performing in-depth exploratory analyses with the help of interactive graphics. In contrast to other software tools that usually aim to visualize the information of the different data types within a single graphic, we display each data type in its own graphic and link them using interactive graphics. Each graphic corresponds to the usual visualization of the corresponding data type and can easily be interpreted. Combining these dynamic graphics by linking, so that objects selected are highlighted in all other graphics, and providing unsupervised statistical methods enables users to perform very effective exploratory analyses. The proposed software does not compete with usual software approaches that offer inferential statistics, but provides a complementary analytical approach. The advantage of our exploratory software regarding the analysis of high-dimensional integrated data sets is demonstrated by an analysis of data collected from acute myeloid leukemia (AML) patients.

## Implementation

To ensure portability and platform independence, SEURAT has been written in Java. Most of the GUI elements are based on JAVA Swing packages so that SEURAT has a uniform look and feel independent of the underlying platform. The software establishes a connection to the R statistical software [5] via Rserve [6]. Rserve is a TCP/IP server which allows other programs to communicate with R. This connection potentially provides access to all functions implemented in R and Bioconductor [7]. For clustering and seriation algorithms SEURAT uses the facilities of the R-packages *amap *[8], *seriation *[9] and *biclust *[10]. In order to use SEURAT, R, the relevant R packages, and the Java Runtime Environment (JRE) 1.6 need to be installed on the user's computer. The software focuses on performing exploratory, visual analyses. To simplify the data import all datasets are assumed to be preprocessed and being in tab-delimited ASCII form. Preprocessing includes the data management and quality control of the different microarray data as well as the normalization, gene filtering and annotation of the data. SEURAT was tested with different data sets, and works well with both data from custom two color gene expression arrays (Stanford 40 k DNA microarrays) and CGH arrays (2.8 k BAC/PAC microarrays) as well as with Affymetrix exon (GeneChip Human Exon 1.0 ST Arrays) and SNP arrays (Genome-Wide Human SNP Arrays 6.0). The preprocessing was performed using R and Bioconductor. For the preprocessing of the exon arrays and for extracting raw copy numbers from the SNP array data we used statistical methods available within the R package *aroma.affymetrix *[11,12]. To extract the genomic regions showing the same genomic variations from array CGH and SNP data we applied the GLAD (Gain and Loss Analysis of DNA) algorithm [13]. This algorithm is available within the Bioconductor package *GLAD *as well as within the R package *aroma.affymetrix*. Alternatively other methods could also be used within this context, e.g. a hidden Markov model approach [14] or the fast binary segmentation algorithm [15]. Additional annotations not available from the Affymetrix annotation files have been added by using the capabilities of BioMart that are accessible with the Bioconductor package *biomaRt *[16]. Detailed R scripts describing each step of the preprocessing are available at the project website.

## Results and Discussion

**Figure 1:**
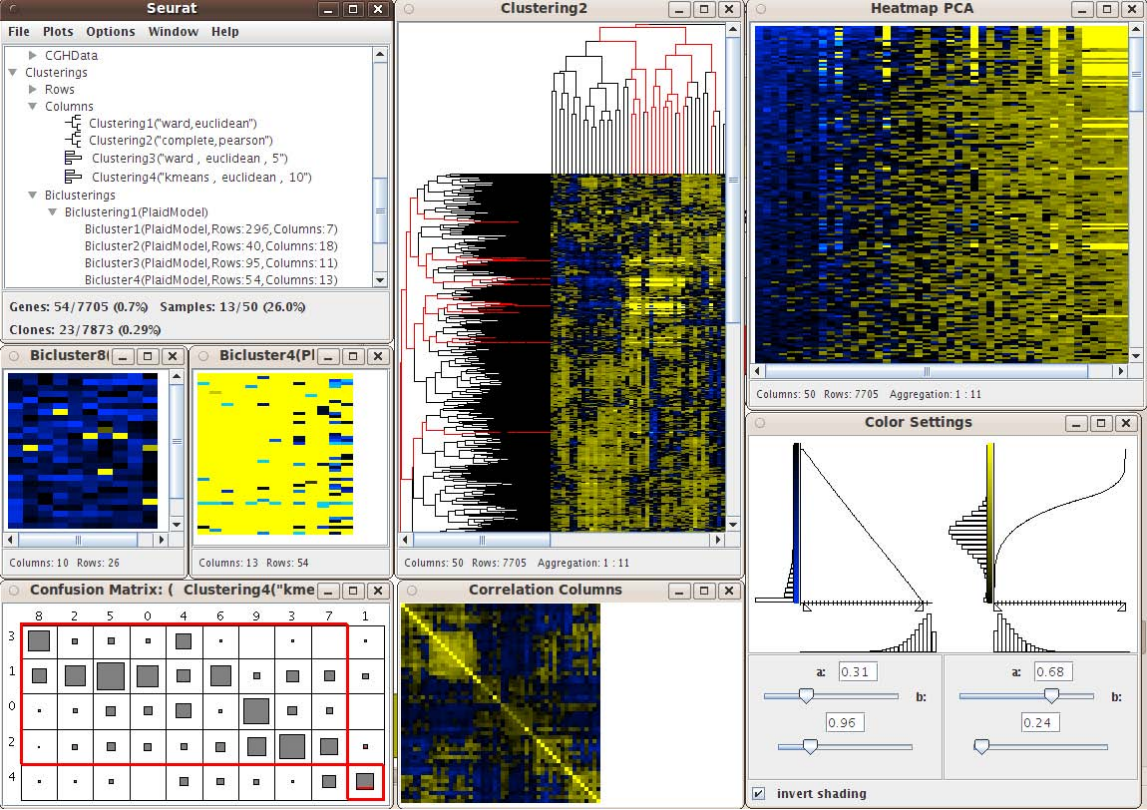
**Screen capture of an unsupervised analysis with SEURAT**. The screenshot displays the data manager and heatmaps showing the results of different unsupervised methods, e.g. a heatmap with dendrograms that displays the result of hierarchical clustering and a heatmap that shows the result of a seriation generated by PCA. Furthermore the color settings menu, a correlation matrix and a confusion matrix are shown. Two resulting biclusters are visualized by heatmaps. 'Bicluster4' is selected and the corresponding genes and samples are highlighted in all other graphics.

**Figure 2:**
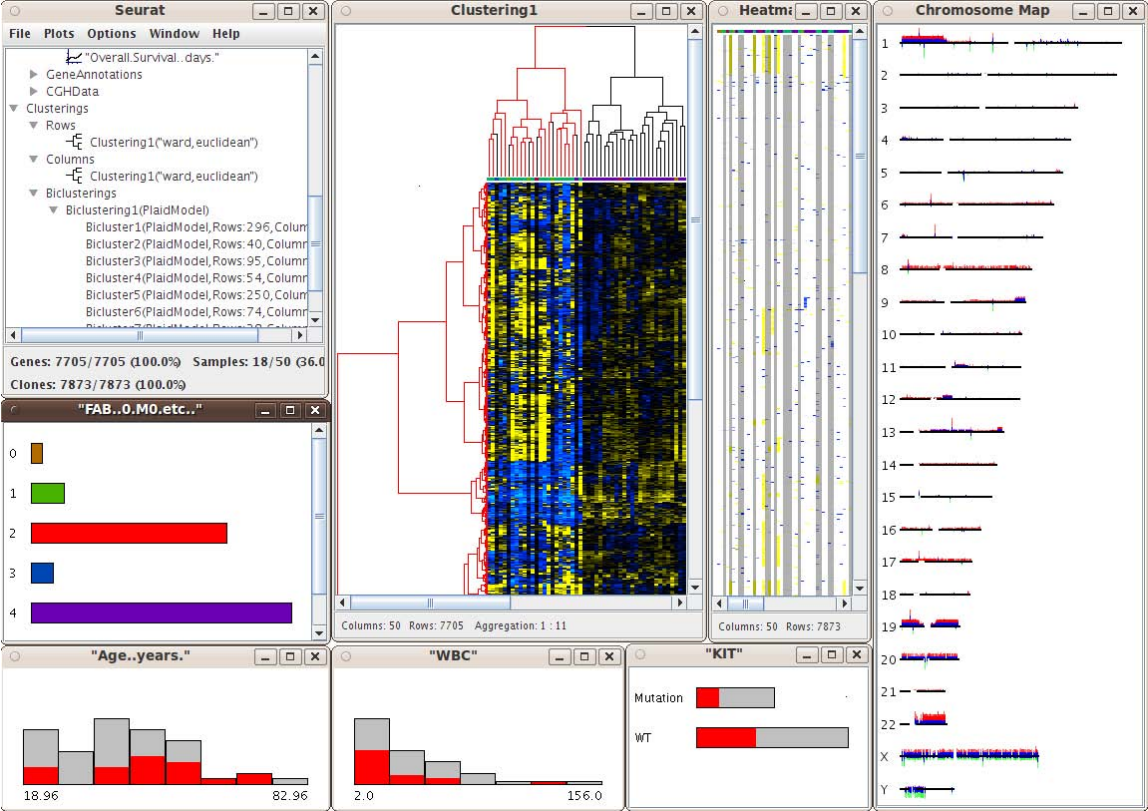
**Interactive graphics example**. Amongst the windows shown are the data manager, a heatmap with dendrograms, a heatmap showing genomic states, a chromosome browser, histograms and barcharts. Colors have been assinged to the barchart showing the categorical variable FAB (French American British classification). This color code is also visible in the first row of the heatmap. The red colored bar of the barchart indicates that the second FAB class is selected and the corresponding patient samples are highlighted in all other graphics.

**Figure 3:**
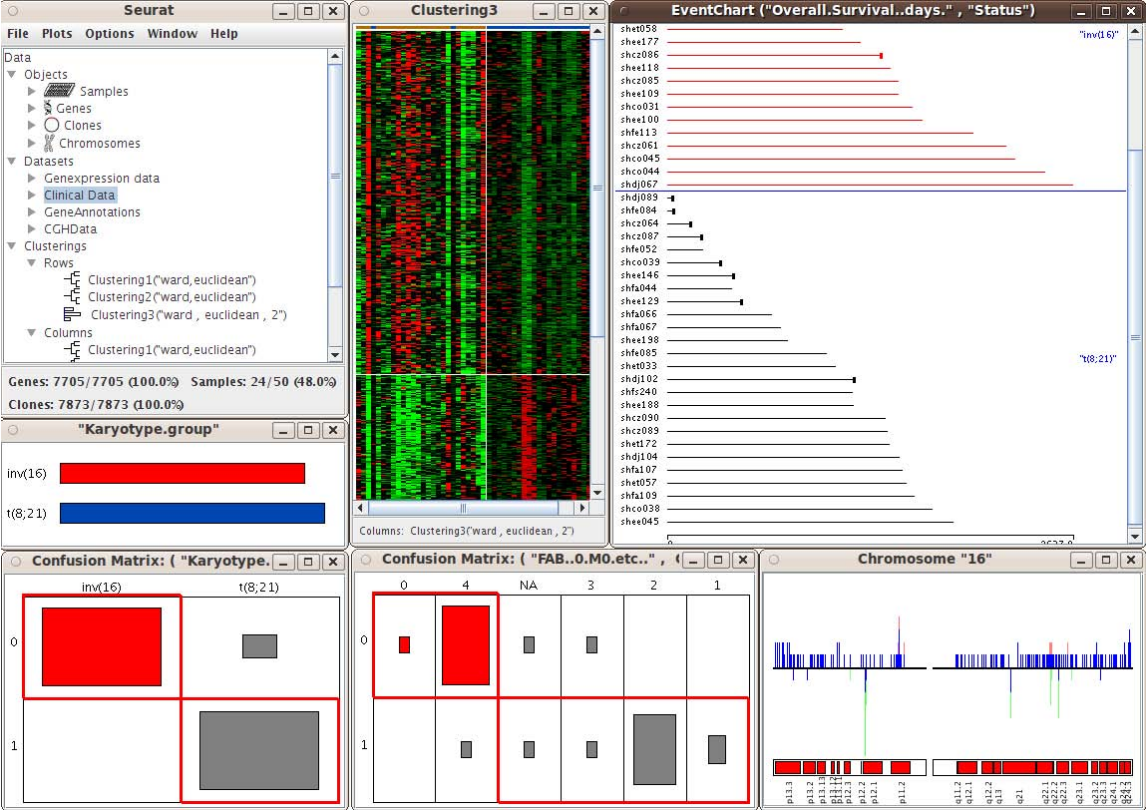
**AML subgroup analysis**. Hierarchical clustering revealed gene expression profiles that strongly coincide with AML subgroups according to secondary genetic aberrations and the FAB classification. The agreement between the clustering and the classification with respect to genetic aberations (inv(16) and t(8,21)) and the FAB (French American British) classification is displayed by confusion matrices. The overall survival times of the subgroups are shown in an eventchart. The chromosome view displays the relative frequencies of genomic variations located at chromosome 16.

The SEURAT software tool is designed to carry out interactive analysis of complex integrated datasets. At present, SEURAT can handle gene expression data with additional gene annotations, clinical data and genomic copy number information arising from array CGH or SNP arrays. The SNPs and array CGH clones need to be mapped to the corresponding genes and therefore the user has to provide the chromosome name and the nucleotide positions within the respective data sets. Detailed information about the supported data formats as well as a detailed instruction how to align the various data can be found in the documentation at the project website. In addition, SEURAT offers a data loading settings menu item that enables the user to specify the columns holding the required information for each data set. To keep track of the different data sets loaded into the workspace, SEURAT provides a data manager, which organizes the data into different, related objects: genes, samples, CGH clones, SNPs and chromosomes. The data manager window is organized as a tree view. Users can browse through the branches of the tree showing datasets and objects and access the gene annotations and clinical data. The gene expression matrix is visualized by a heatmap, where the gene expression levels are represented by colors. This completely interactive heatmap occupies a central position in SEURAT and is the starting point for exploratory analyses. To reorder the gene expression matrix, the user can choose from different clustering and seriation algorithms. Until know SEURAT provides agglomerative hierarchical clustering and k-means clustering and for both of these clustering methods several distance functions are available. If interest lies in an ordering of the gene expression matrix rather than finding distinct clusters, an ordering of the genes and samples using one of a number of seriation algorithms can be carried out. Seriation algorithms are heuristic procedures that try to find an approximately optimal ordering of a set of objects given a loss or merit function. Both seriation algorithms and clustering algorithms belong to the field of combinatorial data analysis. In this context the optimal order of the leaf nodes of a dendrogram resulting from hierarchical clustering can be interpreted as the seriation result. Principle component analysis and multidimensional scaling are established dimension reduction techniques for the analysis of high-dimensional microarray data. Within the seriation algorithms SEURAT provides seriation methods that use the first principle component of a PCA or the first MDS dimension to produce an optimal ordering. In order to reveal subsets of genes coregulated only within a subset of patients SEURAT offers several biclustering algorithms. Biclustering is the simultaneous clustering of rows and columns of a data matrix. Ordinary one-way clustering algorithms cluster objects using the complete feature space, e.g. a clustering of the genes with respect to the gene expression values of all patients. Biclustering algorithms take into account that correlations between genes may only be present for a subset of patients and vice versa. In addition, biclustering algorithms do not assign all objects to a cluster and depending on the biclustering algorithm resulting biclusters are allowed to overlap. Genes that are members of more than one bicluster may be regarded as being involved in more than one biological process. A more detailed description of the different unsupervised statistical methods available within SEURAT can be found at the project website. Clustering and seriation results as well as single biclusters are visualized within an interactive heatmap and in the case of hierarchical clustering, interactive dendrograms can also be displayed (Figure 1). For each heatmap additional functions are available, including functions to change the color and pixel settings and functions to display the correlation matrices of the genes and samples. The results of applying unsupervised methods are added to the tree view within the data manager window so that the user can access the results later in the analysis. To check the quality of different clusterings, clustering results can be compared with a so called confusion matrix plot. This graphic can also be used for comparing a clustering of patients-samples with any categorical clinical variable. The confusion matrix displays objects falling into the same clusters by rectangles with sizes proportional to the number of objects and offers a sorting algorithm to assess the agreement between different clusterings graphically. Clinical data and available gene annotations can be selected within the data manager window. SEURAT automatically distinguishes between categorical and continuous data and the associated information is presented in either interactive barcharts or histograms (Figure 2). Additional functions allow the user to change the bin-width of the histograms, or reorder the barcharts according to different criteria. It is possible to assign colors to the classes of a categorical variable displayed by a barchart and these colors are also added to the heatmap. Available pathway information like GO terms can be inspected by barcharts. For many clinicians some of the most interesting clinical data collected are survival times and other time to event data. Often, interest lies in how time to event data is related to certain gene expression patterns or genomic variations. In SEURAT time to event data can be visualized by so called eventcharts [17]. Unlike commonly used survival plots such as the Kaplan-Meier curve which show aggregate information in the form of estimated survival probabilites over time, the eventchart displays the raw data at the individual level. The use of Kaplan-Meier curves is not practical within SEURAT, because it is not possible to reasonably connect such a graphic bidirectionaly with other linked graphics. Eventcharts display each individual observation by horizontal lines and this representation is more suited for the framework of interconnected graphics. When dealing with time to event data, possible censoring has to be considered. To display observed events within the eventcharts a small vertical bar is drawn at the end of the horizontal line. A missing bar indicates that the event of interest has not been observed and thus the observation time is censored. As an additional feature it is possible to reorder and group the horizontal time lines according to other clinical variables. Preprocessed array CGH data and SNP array data holding information about cytogenetic gains and losses can be displayed like gene expression data in a heatmap and can also be explored with a chromosome browser tool. The chromosome browser provides a global view of all chromosomes of the human genome. The relative frequencies of gains and losses are visualized by barcharts along the chromosomes. In addition each chromosome can be explored in a larger individual plot in which cytoband information is also interactively displayed. All the graphics described are linked with one another, so that any transient selection of objects in one graphic will result in highlighting of the corresponding objects in all other associated graphics. SEURAT provides printing functionality and thus it is possible to export all graphics as pdf files. Testing SEURAT on genomics data derived from acute myeloid leukemia (AML) patients demonstrated the power of this novel analysis tool. Here, the interactive graphics allowed a much faster evaluation of the data enabling the translational researcher to come to meaningful results in a more reasonable time. Furthermore, the interactive graphics and interconnection with clinical data easily allowed the detection of leukemia subgroups of potential biological relevance such as subgroups of AML cases harboring distinct gene expression profiles based on similar underlying secondary genetic aberrations (e.g. AML cases with an inv(16) and an additional trisomy 8; Figure 3).

## Conclusions

SEURAT is a new software tool which is capable of integrated analysis of gene expression, array CGH and SNP array and clinical data using interactive graphics. The focus of SEURAT is on exploratory analysis that enables biological and medical experts to uncover new relations in high-dimensional biological and clinical datasets and thus supports the process of hypothesis generation. To our knowledge, no other software that aims to perform integrated analysis of microarray data offers such a high level of interactivity. The concept of combining many interactive graphics by logical linking and the broad spectrum of unsupervised methods is unique. Because of the object oriented design of the software it will be possible to add additional graphics like parallel coordinates with interactive capability. In addition, with the use of Rserve, the complete functionality of R and Bioconductor is available to include more statistical methods in SEURAT. In particular, further clustering algorithms (e.g. model-based clustering) will be investigated for adoption in later versions of SEURAT. In the future, we plan to adapt SEURAT to integrate other microarray based data types such as loss of heterozygosity data, also available from SNP arrays, as well as information from protein arrays or epigenetic data arising from methylation arrays. While this will further improve SEURAT, the current version already provides a powerful means for the integrative and interactive analysis of complex genomics data sets. Therefore, SEURAT will likely contribute to refined insights into cancer biology such as acute myeloid leukemia.

## Availability and requirements

**Project name**: SEURAT

**Project home page**: http://seurat.r-forge.r-project.org/

**Operating system(s)**: Platform independent

**Programming language**: Java and R

**Other requirements**: Java 1.6 or higher, R 2.8 or higher, R-packages: Rserve, amap, seriation and biclust License: GNU GPLv3

**Any restrictions to use by non-academics**: None

## Abbreviations

AML: acute myeloid leukemia; ASCII: American Standard Code for Information Interchange; BAC/PAC: bacterial artificial chromosome/P1-derived artificial chromosome; CGH: comparative genomic hybridization; FAB classification: French American British classification; GUI: graphical user interface; inv(16): inversion mutation at chromosome 16; JRE: Java Runtime Environment; RNAi: RNA interference; SNP: single nucleotide polymorphism; TCP/IP: Transmission Control Protocol/Internet Protocol; t(8,21): translocation mutation between chromosome 8 and 21.

## Competing interests

The authors declare that they have no competing interests.

## Authors' contributions

LB, AB and AU conceived the software. AG was responsible for the software architecture and implementation. MS, SL, LB, AB, and AU were involved in designing and testing the software. MS preprocessed and annotated the example data sets and wrote the documentation. FR and KD collected the clinical and molecular data. MS wrote the manuscript and all authors revised and approved the final manuscript. AG and MS contributed equally.

## Pre-publication history

The pre-publication history for this paper can be accessed here:

http://www.biomedcentral.com/1755-8794/3/21/prepub
